# Effects of vasodilating medications on cerebral haemodynamics in health and disease: systematic review and meta-analysis

**DOI:** 10.1097/HJH.0000000000002033

**Published:** 2018-12-11

**Authors:** Alastair J.S. Webb

**Affiliations:** Centre for Prevention of Stroke and Dementia, John Radcliffe Hospital, University of Oxford, Oxford, UK

**Keywords:** cerebral arterial pulsatility, cerebrovascular reactivity, hypertension, stroke, vasodilator

## Abstract

**Objectives:**

Vasodilating antihypertensives prevent stroke and potentially cerebral small vessel disease but their effects on cerebrovascular haemodynamics beyond blood pressure lowering are unclear.

**Methods:**

We searched PubMed, Medline, Embase, Cinahl, Psychinfo, Health Business Elite and Health Management Information Consortium for randomized studies of vasodilating medications, compared to no treatment or nonvasodilators, that reported effects on cerebral blood flow (CBF), mean blood flow velocity (MFV) or cerebrovascular reactivity. Absolute and standardized mean differences (SMD) were combined by inverse-variance weighted fixed or random-effects meta-analysis stratified by study design, population characteristics and vasodilator class.

**Results:**

In 35 studies reporting 57 comparisons, there was a reduction in SBP (−4.13 mmHg, −7.55 to −0.71, *P* = 0.018) but no change in MFV (ΔMFV 1.11, confidence interval −0.93 to 3.14, *P* = 0.29, 23 comparisons). MFV increased in patients with underlying conditions (3.41, 0.24 to 6.57, *P* = 0.04) but not in healthy study participants (−1.27, −5.18 to 2.64, *P* = 0.68), with no differences by vasodilating drug class. Cerebral pulsatility index was reduced across all studies (Δ pulsatility index −0.04, −0.07 to −0.02, *P* = 0.001; Δ pulsatility index -SMD −0.32, −0.47 to −0.16, *P* < 0.001), except in studies reporting responses to single drug doses (Δ pulsatility index 0.00, −0.09 to −0.08, *P* = 0.93). Despite evidence of reporting and publication bias, there was an apparent consistent reduction in CBF with vasodilators (CBF-SMD −0.24, −0.46 to −0.02, *P* = 0.03) with a significant increase in cerebrovascular reactivity-SMD (0.48, 0.13–0.83, *P* = 0.007).

**Conclusions:**

Despite reducing SBP, vasodilators did not significantly impair absolute CBF but improved cerebrovascular pulsatility and reactivity, suggesting therapeutic potential in preventing stroke and cerebral small vessel disease.

## BACKGROUND

Blood pressure (BP)-lowering treatment significantly reduces the risk of recurrent stroke [1] and probably reduces cerebral small vessel disease [2]. Vasodilating antihypertensive medications (calcium channel blockers, angiotensin receptor blockers) appear to be more effective in prevention of cerebrovascular disease than vasoconstricting antihypertensives (β-blockers) [1,3,4], despite similar effects on brachial BP. Furthermore, antiplatelet medications with pleiotropic vasodilating actions (cilostazol [5], dipyridamole [6]) appear to reduce recurrent stroke more than expected from their antiplatelet effects alone. These differences may be because of systemic effects on BP variability [1] or central aortic BP [7], but could reflect the transmission of systemic haemodynamic effects to the cerebral circulation [8] or direct effects of treatment on cerebrovascular haemodynamics [9].

Previous studies and systematic reviews have assessed the effect of single drugs or single cerebrovascular indices, whether static measures of resting blood flow [cerebral blood flow (CBF); mean blood flow velocity (MFV) [10]] or functions of cerebral vessels [cerebral arterial pulsatility; [9,11] cerebrovascular reactivity (CVR); to carbon dioxide (CO_2_) [12,13]]. However, studies have been underpowered, have not meta-analyzed results because of insufficient studies with significant heterogeneity and have focused on specific conditions, drugs or specific physiological measures, limiting the ability to draw conclusions about the effects of vasodilatation across patient groups. However, maintenance of a sufficient supply of blood to the parenchyma depends upon resting blood flow, consistent perfusion throughout the cardiac cycle and the capacity to adapt to environmental challenges. Therefore, a valid assessment of the potential of vasodilating agents to improve cerebrovascular outcomes depends upon measuring their effects on multiple functions in both health and disease.

This study therefore assessed whether there was evidence for a consistent effect of vasodilating medications across patient groups, on both resting blood flow and dynamic cerebrovascular functions.

## METHODS

### Search strategy

PubMed, Medline, Embase, Cinahl, Psychinfo, Health Business Elite and Health Management Information Consortium were searched between inception and 21 June 2018 to identify controlled trials comparing effects of vasodilating treatments with non-vasodilating medication, placebo or no change in treatment. Accepted outcomes included effects on CBF, CBF velocity or CVR, according to a prespecified search strategy and inclusion criteria. Study titles, potential abstracts and full-text articles were reviewed sequentially (PRISMA flowchart, Supplemental Figure 1). All included studies were assessed for quality according the Cochrane manual [14]. Study characteristics, population demographics, methods, intervention, dose, duration of treatment, cerebrovascular indices and BP were extracted.

### Analysis

Studies were categorized by treatment allocation, population characteristics (healthy vs. disease), treatment within 7 days (for populations with an acute stroke), single dose vs. ongoing treatment and crossover vs. parallel group design. Outcome measures included peak systolic (PSV), MFV, end diastolic velocity (EDV) and Gosling's pulsatility index [(PSV – EDV)/MFV] on transcranial ultrasound. For studies reporting mean CBF on MRI, computed tomography (CT) or other perfusion-based imaging methods, mean CBF and the SD of CBF at follow-up were converted into the standardized mean difference (SMD) between treatment groups (difference in means/pooled SD), and transformed to normalize effect size distribution (Hedge's g). Responses to CVR tests (breath-holding, inhaled CO_2_ 5–8%, acetazolamide challenge or the response to hyperventilation) were expressed as SMD for percentage change in CBF or MFV after a vasodilating stimulus compared with the prestimulus measurement (Hedge's g).

Comparisons were combined in meta-analyses by the difference of absolute mean values at follow-up for TCD studies and by the SMD of effects at follow-up for CBF and CVR, weighted by the inverse variance [15]. For meta-analyses combining absolute measures, the inverse variance of comparisons that did not report the SD at follow-up were imputed from the ratio of the study size to the size of all studies reporting the SD at follow-up. Sensitivity analyses were performed including only studies reporting the SD at follow-up. Studies reporting comparisons of more than one vasodilator vs. a single control group were included as separate comparisons, but only including the number of individuals from one arm for estimation of the inverse variance in imputed values or in calculation of the inverse variance of the SMD. Sensitivity analyses were performed with the inverse variance of the studies divided by the number of comparisons with the same control group. Crossover studies were included as individual comparisons without adjustment for intra-group correlation because of the lack of available data, but sensitivity analyses were performed stratifying effects by crossover design.

The principal outcomes were the effect of treatment with a vasodilating medication compared to a non-vasodilating medication, placebo or no change in treatment, on ΔMFV, Δ pulsatility index, ΔCBF, or reactivity to CO_2_, with secondary outcomes on PSV or EDV, SBP, DBP and pulse pressure. As few studies reported effects on pulse pressure, study-specific estimates were generated from the difference in group mean SBP and DBP. Analyses were stratified by study design, comparisons with antihypertensives or no active treatment as the control group, by the active drug class, by method of measurement of CBF or reactivity to CO_2_, by population demographics, disease duration and acuity of treatment.

To assess the presence of treatment interactions with study design, treatment acuity or population characteristics, determinants of effect size (SMD or absolute differences) and their interactions with treatment were identified from general linear models, weighted by the inverse variance.

## RESULTS

The systematic search returned 10 342 titles, of which 478 potentially eligible abstracts were reviewed and 105 studies were reviewed in full. Of 36 eligible studies, two studies reported results from the same population. Of the remaining 35 studies, 20 reported effects on blood flow velocity on TCD, 17 reported effects on CBF and 10 studies reported effects on CVR, with a total of 57 comparisons between vasodilators and non-vasodilators across all studies and methods (PRISMA flowchart, Supplemental Figure I).

The majority of studies included were small (median 24.5 participants, interquartile range 16.5–47.75) with only three studies including more than 100 participants. Study quality was poor with only half of 35 studies being at low risk of bias in each category of the quality assessment (Supplemental Figure 2). Furthermore, although no studies significantly exceeded expected confidence limits (Supplemental Figure 2), there was evidence of publication bias in the assessment of CBF and reactivity outcomes, with a bias toward positive results. However, this may have reflected erroneous assignment of the direction of effect in one study (where the numerical data differed from the text description) distorting the distribution of results and without which there was a homogeneous, positive effect of treatment [16]. There was no evidence of publication bias in studies reporting effects on MFV and pulsatility index on TCD (Supplemental Figure 2).

**FIGURE 1:**
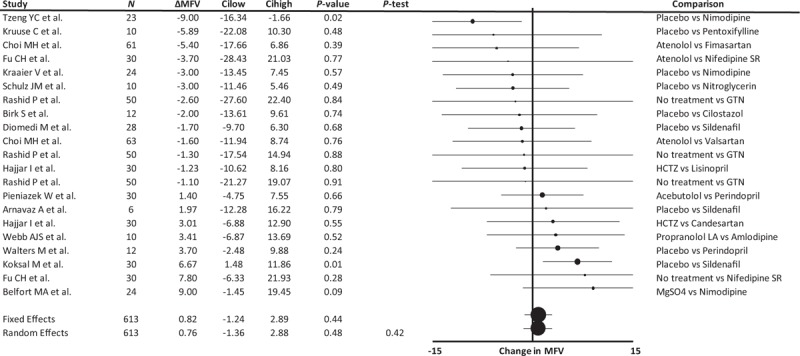
Forest plot of difference in mean flow velocity between groups randomized to a vasodilating medication vs. a non-vasodilating control group. Control groups included patients treated with either placebo, a non-vasodilating medication or no change in treatment, with effect sizes combined by both fixed effects and random effects meta-analysis weighted by the inverse variance. ΔMFV, change in mean flow velocity; Cilow, confidence interval lower limit; Cihigh, confidence interval upper limit; GTN, glyceryl trinitrate; HCTZ, hydrochlorothiazide; *N*, number of study participants; *P*-het, *P* value for heterogeneity.

In total, 17 studies reported no significant effect in 23 comparisons of vasodilators with control groups on cerebral MFV, with only a small, non-significant increase in MFV and minimal heterogeneity between studies (Fig. 1). Differences in MFV between studies were not explained on stratification by an active vs. placebo control group (Supplemental Figure 3), by crossover vs. parallel group design or by whether a single dose or prolonged treatment was given. However, although there was no significant change in MFV in studies in healthy individuals, in limited studies of patients with an underlying condition there was a non-significant decrease in MFV in patients treated in the acute phase of a cerebrovascular event compared to a significant increase in MFV in patients treated in the chronic phase of an illness. In particular, MFV increased in two studies in which vasodilators were initiated against a background of untreated hypertension [17] or preeclampsia [18] (Supplemental Figure 4). In a stepwise, general linear model weighted by the inverse variance, vasodilator medication (*P* = 0.026), acuity of treatment (*P* = 0.037) and healthy vs. underlying disease (*P* = 0.028) independently predicted difference in absolute MFV between groups. Overall, the class of vasodilator medication explained a proportion of the variance in the meta-analysis, there were no significant differences between specific drug classes (Supplemental Figure 5).

**FIGURE 2:**
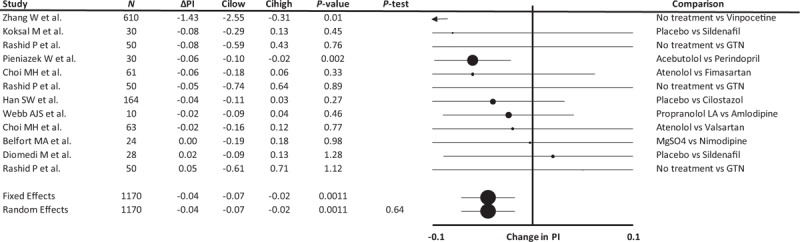
Forest plot of difference in cerebral arterial pulsatility index between groups randomized to a vasodilating medication vs. a non-vasodilating control group. Control groups included patients treated with either placebo, a non-vasodilating medication or no change in treatment, with effect sizes combined by both fixed effects and random effects meta-analysis weighted by the inverse variance. All studies report the MCA pulsatility index except for Zhang *et al.*[19] who reported basilar artery pulsatility index. ΔPI, change in mean flow velocity; Cilow, confidence interval lower limit; Cihigh, confidence interval upper limit; GTN, glyceryl trinitrate; *N*, number of study participants; *P*-het, *P* value for heterogeneity.

Assignment to a vasodilator was associated with a reduction in middle cerebral artery (MCA) pulsatility (Fig. 2), both as the absolute difference in MCA-pulsatility index and as a SMD (−0.32, −0.47 to −0.16, *P* < 0.001, *P*-heterogeneity 0.82). There was no significant difference on stratification by active vs. no active control, healthy vs. underlying conditions or acute vs. chronic treatment (Supplemental Figure 7). The largest study [19] selectively reported effects on differences in basilar artery pulsatility index, without reporting effects on MCA-pulsatility index, and had the most extreme point estimate with wide confidence intervals. Exclusion of this study had no significant impact on the result (absolute Δ pulsatility index: −0.04, −0.07 to −0.02, *P* < 0.001, *P*-het 0.98; SMD Δ pulsatility index: −0.32, −0.60 to −0.04, *P* = 0.024, p-het 0.74). In limited studies, there was a significant increase in PSV and a smaller, non-significant increase in EDV (please see Supplemental Figure 6), although the small number of small studies limits the reliability of the analysis.

**FIGURE 3:**
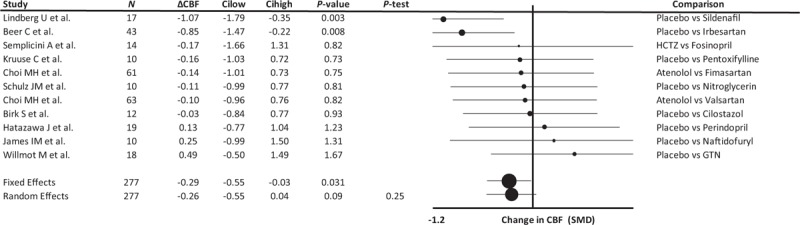
Forest plot of difference in cerebral blood flow between groups randomized to a vasodilating medication vs. a non-vasodilating control group. Control groups included patients treated with either placebo, a non-vasodilating medication or no change in treatment, with effect sizes combined by both fixed effects and random effects meta-analysis weighted by the inverse variance. Differences between groups are expressed as the SMD. ΔCBF, change in cerebral blood flow velocity; Cilow, confidence interval lower limit; Cihigh, confidence interval upper limit; GTN, glyceryl trinitrate; HCTZ, hydrochlorothiazide; *N*, number of study participants; *P*-het, *P* value for heterogeneity; SMD, standardized mean difference.

Treatment with vasodilators was associated with a small reduction in CBF across all studies (Fig. 3), but this was principally driven by two outliers that used newer MRI perfusion or CT perfusion techniques and were responsible for all the heterogeneity in the analysis. One reported a small difference in CBF but with an unusually low SD that distorted the SMD [20] and the other reported a large difference in CBF (21%), evident at 30 days but not at 3 days [21]. Combining all other studies showed a consistent, null effect of treatment on CBF (0.00, −0.31 to 0.32, *P* = 0.32, *P*-heterogeneity 0.99, nine comparisons). Study design, measurement method, acuity of treatment, duration of treatment, population characteristics and allocated medication had no interaction with treatment effects on CBF either on stratified meta-analyses (please see Supplemental Figure 8) or in an inverse-variance weighted linear model.

**FIGURE 4:**
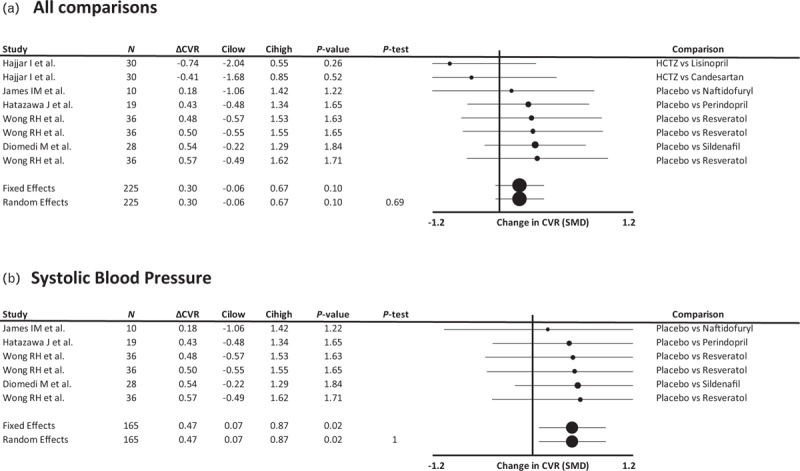
Forest plot of difference in reactivity of cerebral blood flow or blood flow velocity to a CO_2_ challenge between groups randomized to a vasodilating medication vs. a non-vasodilating control group. Control groups included patients treated with either placebo, a non-vasodilating medication or no change in treatment (A) or either placebo or no change in treatment (B), with effect sizes combined by both fixed effects and random effects meta-analysis weighted by the inverse variance. Differences between groups are expressed as the SMD. ΔCVR, change in cerebral vascular reactivity; Cilow, confidence interval lower limit; Cihigh, confidence interval upper limit; HCTZ, hydrochlorothiazide; *N*, number of study participants; *P*-het, *P* value for heterogeneity; SMD, standardized mean difference.

Effects of vasodilators on the SMD in CVR demonstrated a non-significant increase when including all studies reporting sufficient data. However, there was a single outlier study (Supplemental Figure 2) reporting two comparisons [16] with a common, active control group (hydrochlorothiazide), with a potential reporting error. Only including studies with an inactive control demonstrated a consistent, significant increase in CVR with vasodilating medications, without heterogeneity (Fig. 4). The three other studies identified in the systematic search [20,22,23] that reported CVR results, which were not compatible with the meta-analysis, also demonstrated an increase in CVR with vasodilators. There were too few comparisons to compare medications, populations or study designs.

**FIGURE 5:**
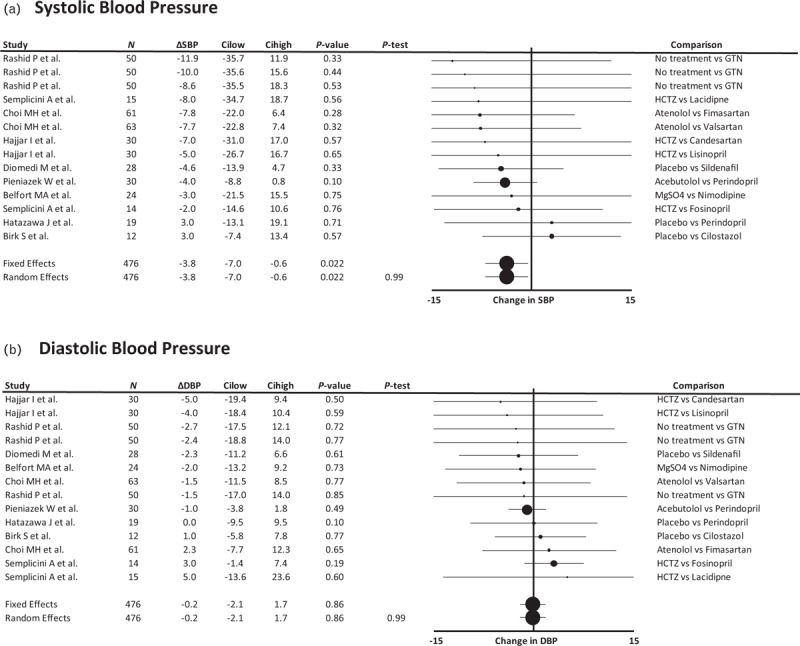
Forest plot of difference in SBP and DBP between groups randomized to a vasodilating medication vs. a non-vasodilating control group. Control groups included patients treated with either placebo, a non-vasodilating medication or no change in treatment, with effect sizes combined by both fixed effects and random effects meta-analysis weighted by the inverse variance. Differences between groups are expressed as the SMD. Cilow, confidence interval lower limit; Cihigh, confidence interval upper limit; GTN, glyceryl trinitrate; HCTZ, hydrochlorothiazide; *N*, number of study participants; *P*-het, *P* value for heterogeneity; SMD, standardized mean difference.

Vasodilator medications consistently reduced SBP across studies but did not change DBP (Fig. 5) or MFV. Group level estimates of pulse pressure showed a non-significant reduction in pulse pressure with vasodilators (Supplemental Figure 9). There was still a consistent reduction in pulsatility index in these studies (Supplemental Figure 10). There was no significant relationship between effects on SBP or DBP and the degree of change in MFV or pulsatility index (Supplemental Figure 11). There were too few studies reporting effects on BP and either CBF or CVR to determine relationships between change in BP and change in CBF or reactivity.

## DISCUSSION

This study demonstrated an improvement in cerebral arterial pulsatility and CVR to CO_2_ with vasodilating medications, with minimal or no effect on resting CBF despite a reduction in SBP. These effects were consistent across populations and drug classes, although there were too few studies of sufficient power to draw reliable conclusions about subgroups. However, there was evidence of study design-related, reporting and publication bias.

Increased MCA pulsatility index [8,24] and reduced CVR [12] have been associated with more severe small vessel disease and a potentially increased risk of acute cerebrovascular events. This may reflect a causative factor because of barotrauma from systolic pressure waves, hypoperfusion because of low CBF in diastole or an impaired capacity of the cerebrovasculature to adapt to sustained BP changes [8]. However, in the absence of reliable longitudinal studies or interventional trials, it is unknown if these physiological changes are directly causative, secondary to established injury or bystander associations reflecting a common underlying process. By identifying that vasodilators improve cerebrovascular dynamic function without affecting resting blood flow, we can test whether these physiological mechanisms are causative. As vasodilators disproportionately affected SBP over DBP, with a non-significant trend to a reduction in study level estimates of pulse pressure, the resulting reduction in cerebral pulsatility may reflect either systemic or central effects, although effects on CVR would be expected to reflect central effects. However, either mechanism would represent a specific treatment option beyond mean BP lowering to prevent stroke and reduce progression of cerebral small vessel disease.

The lack of a clinically significant decrease in resting CBF and MFV with vasodilator treatment, despite a reduction in SBP, is consistent with the conclusions of previous studies [10] and the expected effect of chronic interventions in patients if cerebral autoregulation is intact. This was consistent across subgroups, with only a small increase in MFV in patients with chronic underlying conditions (hypertension, ischaemia and cognitive impairment), potentially implying an improvement in underlying vasoconstriction. This supports the well-tolerated use of vasodilators in chronic cerebrovascular disease, consistent with the results of large randomized controlled trials [25]. However, there in the acute phase of stroke study there was insufficient evidence regarding the effects of vasodilators, with a trend to a reduction in MFV and no improvement in pulsatility index with acute dosing. Recent clinical trials of vasodilators in the acute phase of stroke have also demonstrated null effects (glyceryl trinitrate in the Efficacy of Nitric Oxide in Stroke study; [26] candesartan in Scandinavian Candesartan Acute Stroke Trial [27]), although treatment in the hyperacute phase is currently being assessed (rapid intervention with glyceryl trinitrate in hypertensive stroke trial-2) [28]. Therefore, further studies are required to define the physiological effects of vasodilators in the acute phase of stroke and whether they have the potential for clinical benefit.

The lack of heterogeneity by drug class in these analyses implies that vasodilators act upon a consistent physiological mechanism across populations, regardless of study design, supporting the validity of performing a composite meta-analysis combining treatments and populations. However, it is likely that the preponderance of small studies limited the study's power to identify subgroup differences. Furthermore, some analyses were excessively influenced by outliers reporting a greater-than expected level of accuracy or effect size that may have distorted the results. Nonetheless, the overall consistency of effects across different drug classes and mechanisms of action, including a number of agents with limited clinical data (resveratrol, vinpocetine), suggests that the calculated average effects of vasodilators on each cerebrovascular function are valid.

The systematic review has a number of limitations. First, there were only limited, small studies of variable quality that compared the effects of vasodilating and non-vasodilating medications on cerebral haemodynamics. Therefore, the results are hypothesis-generating and larger studies are required, although the findings are consistent with the clinical cerebrovascular outcomes in large randomized trials of antihypertensive vasodilators. Second, there were too few studies to reliably identify or exclude subgroup differences, limiting assessments of specific drug classes or differences between populations. Third, a number of medications (resveratrol, vinpocetine) have relatively little evidence for the specificity of their pharmacological effect, and could potentially have actions that do not reflect vasodilatation. However, the similarity of their effects compared to established vasodilators in this study supports the validity of their inclusion. Finally, there are no studies comparing the effects of treatment on cerebrovascular haemodynamics with clinical outcomes.

This study adds to the evidence that vasodilators have limited effects on resting CBF but may improve dynamic cerebrovascular functions. This supports their well-tolerated use as antihypertensives in chronic hypertension and secondary stroke prevention, as demonstrated in large clinical trials. However, there was a lack of good quality phase 2 trials investigating the direct physiological impact of vasodilators on the cerebral circulation in different patient groups. In particular, further research is required to assess whether vasodilators not used as antihypertensives (cilostazol, sildenafil, isosorbide mononitrate) have potentially beneficial physiological effects beyond BP lowering, and whether this provides a potential novel treatment option in preventing progression of cerebral small vessel disease or recurrent cerebrovascular events that should be tested in phase 3 clinical trials.

## SUMMARY

This systematic review and meta-analysis demonstrated that in the face of a significant reduction in SBP, vasodilating medications did not significantly impair CBF or blood flow velocity at rest, but had relatively consistent beneficial effects on CVR and cerebral arterial pulsatility. However, the small number of heterogeneous populations, variability in medications tested, and low overall quality of studies demonstrates the need for larger, better designed studies to assess the physiological impact of vasodilating medications of each drug class on the cerebral circulation and their potential for clinical applications beyond BP lowering.

## ACKNOWLEDGEMENTS

A.W. is funded by a Wellcome Trust CRCD Fellowship (206589/Z/17/Z) and a BHF project grant.

### Conflicts of interest

There are no conflicts of interest.

## Supplementary Material

Supplemental Digital Content
