# Supplementary material for: Effects of vasodilating medications on cerebral haemodynamics in health and disease: systematic review and meta-analysis
Source: J Hypertens. 2018 Dec 11;37(6):1119–25. doi: 10.1097/HJH.0000000000002033 (PMC6513078; doi:10.1097/HJH.0000000000002033)
Supplement: Supplemental Digital Content [file jhype-37-1119-s001.docx]

**SUPPLEMENTAL DATA**

**Effects of vasodilating medications on cerebral hemodynamics in health and disease: systematic review and meta-analysis**

Alastair JS Webb DPhil

**Affiliations:** ^†^ Centre for Prevention of Stroke and Dementia, University of Oxford, UK

**Supplemental Figure 1. PRISMA flowchart of identification, screening and inclusion of studies.**

**Abstracts screened**
(n = 478 )

**Records after duplicates removed**
(n = 10342 )

## Identification

## Eligibility

## Included

## Screening

**Comparisons of Reactivity to CO2**
(n = 14)

**Comparisons of CBF**

(n = 20)

**Comparisons of TCD Measures (MFV or PI)**
(n = 32)

**Records excluded**
(n = 373)

**Full-text articles excluded**:

Unobtainable / only abctract published 6

Duplicate Population 6

Not Primary Data 5

No Control Group 22

Wrong patient group 4

No useable data 27

**Full-text articles assessed**
(n = 105)

**Studies included in quantitative synthesis** (meta-analysis)
(n = 35)

**Additional records identified through other sources**
(n = 14 )

**Records identified through database searching**
(n = 14,171)

**Supplemental Figure 2. Assessment of quality of included studies.** A) Assessment of study quality according to Cochrane Manual criteria, assessing each study for a low, unclear or high risk of bias; B – D) funnels plots of the effect size of each comparison against the standard error with 95% confidence limits for change in cerebral blood flow (CBF), change in mean flow velocity (MFV) or change in cerebrovascular reactivity (CVR). SMD – standardised mean difference. SE – standard error


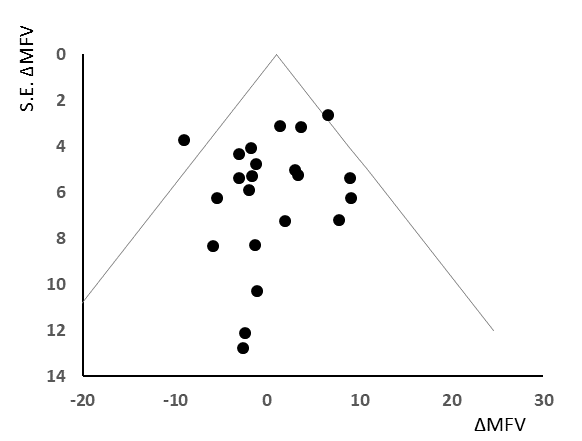

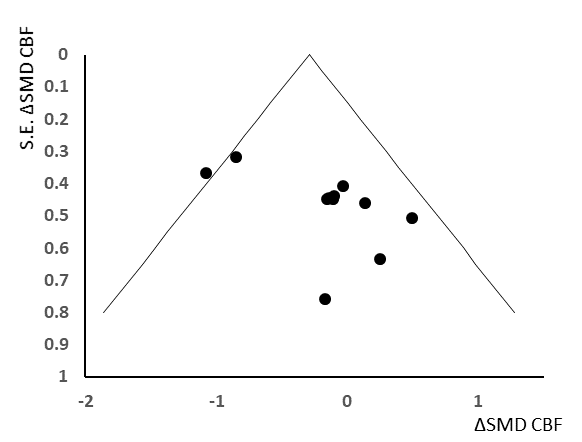

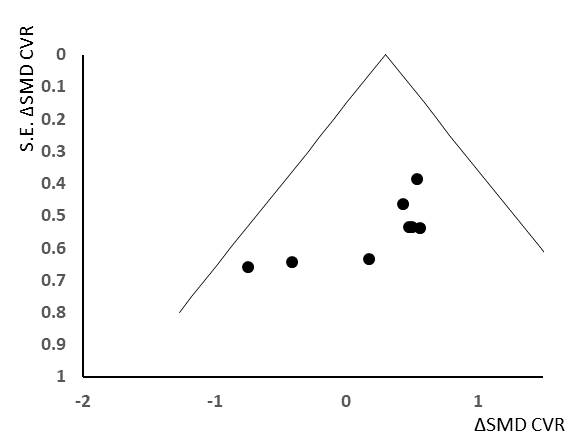

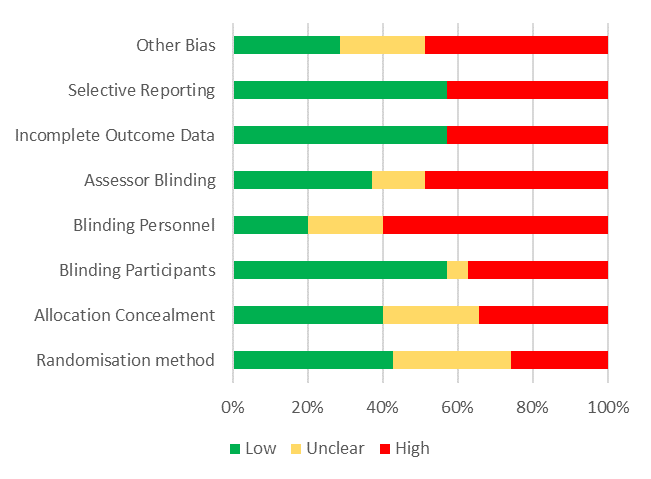


**A)**

**B)**

**C)**

**D)**

**Supplemental Figure 3. Forest plot of difference in MFV between groups randomised to a vasodilating medication versus placebo or no change in treatment.**  Effect sizes are combined by both fixed effects and random effects meta-analysis weighted by the inverse variance. ∆MFV- change in mean flow velocity; Cilow- confidence interval lower limit; Cihigh confidence interval upper limit; p-het – p value for heterogeneity; N – number of subjects; GTN – glyceryl trinitrate;

**Supplemental Figure 4. Forest plots of difference in mean flow velocity between groups randomised to a vasodilating medication versus a non-vasodilating control group, stratified for Healthy Individuals (A), patients treated within 7 days of a stroke or TIA (B) or patients with chronic conditions (C).**  Control groups included patients treated with either placebo, a non-vasodilating medication or no change in treatment, with effect sizes combined by both fixed effects and random effects meta-analysis weighted by the inverse variance. Chronic conditions include previous stroke, cognitive impairment, hypertension or migraine. ∆MFV- change in mean flow velocity; Cilow- confidence interval lower limit; Cihigh confidence interval upper limit; p-het – p value for heterogeneity; N – number of subjects; GTN – glyceryl trinitrate; HCTZ – hydrochlorothiazide;

**A) Healthy Individuals**

**B) Acute cerebrovascular events**

**C) Chronic underlying conditions**

**Supplemental Figure 5. Forest plots of difference in mean flow velocity between groups randomised to a vasodilating medication versus a non-vasodilating control group, stratified by vasodilator drug class.**  Control groups included patients treated with either placebo, a non-vasodilating medication or no change in treatment, with effect sizes combined by both fixed effects and random effects meta-analysis weighted by the inverse variance. Chronic conditions include previous stroke, cognitive impairment, hypertension or migraine. ∆MFV- change in mean flow velocity; Cilow- confidence interval lower limit; Cihigh confidence interval upper limit; p-het – p value for heterogeneity; N – number of subjects; GTN – glyceryl trinitrate; HCTZ – hydrochlorothiazide;

1. **Phosphodiesterase inhibitors**

1. **Calcium Channel Blockers**

1. **Renin-angiotensin system inhibitors**

1. **Nitric Oxide donors**

**Supplemental Figure 6. Forest plot of difference on peak systolic (A) or end diastolic (B)** **velocity between groups randomised to a vasodilating medication versus a non-vasodilating control group.**  Control groups included patients treated with either placebo, a non-vasodilating medication or no change in treatment, with effect sizes combined by both fixed effects and random effects meta-analysis weighted by the inverse variance. ∆MFV- change in mean flow velocity; Cilow- confidence interval lower limit; Cihigh confidence interval upper limit; p-het – p value for heterogeneity; N – number of subjects;

**A) Peak Systolic Velocity**

**B) End Diastolic Velocity**

**Supplemental Figure 7. Forest plot of difference in cerebral arterial pulsatility index between groups randomised to a vasodilating medication versus a non-vasodilating control group, stratified by study design.**  Comparisons are stratified by comparisons with placebo or no change in treatment only (A), healthy participants (B), within 7 days of a stroke or TIA (C), chronic conditions (D), or whether medication was only given once (E) or for multiple days (F). Effect sizes are combined by both fixed effects and random effects meta-analysis weighted by the inverse variance. ∆PI- change in mean flow velocity; Cilow- confidence interval lower limit; Cihigh confidence interval upper limit; p-het – p value for heterogeneity; N – number of subjects; GTN – glyceryl trinitrate;

**A) No active control groups**

**B) Healthy participants**

**C) Acute cerebrovascular events**

**D) Chronic treatment in underlying conditions**

1. **Single dose studies**

**F) Prolonged treatment**

**Supplemental Figure 8. Forest plot of difference in cerebral blood flow between groups randomised to a vasodilating medication versus a non-vasodilating control group, stratified by study design.**  Comparisons are stratified by comparisons with placebo or no change in treatment only (A), whether medications were only given as a single dose (B), for healthy participants (C), within 7 days of a stroke or TIA (D) or in the context of chronic conditions (E). Effect sizes are combined by both fixed effects and random effects meta-analysis weighted by the inverse variance. ∆CBF- change in cerebral blood flow; Cilow- confidence interval lower limit; Cihigh confidence interval upper limit; p-het – p value for heterogeneity; N – number of subjects; GTN – glyceryl trinitrate; SMD – standardised mean difference.

1. **No active control groups**

1. **Single dose studies**
2. **Healthy participants**

1. **Acute treatment after cerebrovascular events**

1. **Chronic treatment in underlying conditions**

**Supplemental Figure 9. Forest plot of difference in grouped mean pulse pressure, between groups randomised to a vasodilating medication versus a non-vasodilating control group.**  Control groups included patients treated with either placebo, a non-vasodilating medication or no change in treatment, with effect sizes combined by both fixed effects and random effects meta-analysis weighted by the inverse variance, estimating the study specific pulse pressure from the group mean SBP and DBP. Cilow- confidence interval lower limit; Cihigh confidence interval upper limit; p-het – p value for heterogeneity; N – number of subjects; GTN – glyceryl trinitrate; HCTZ – hydrochlorothiazide;


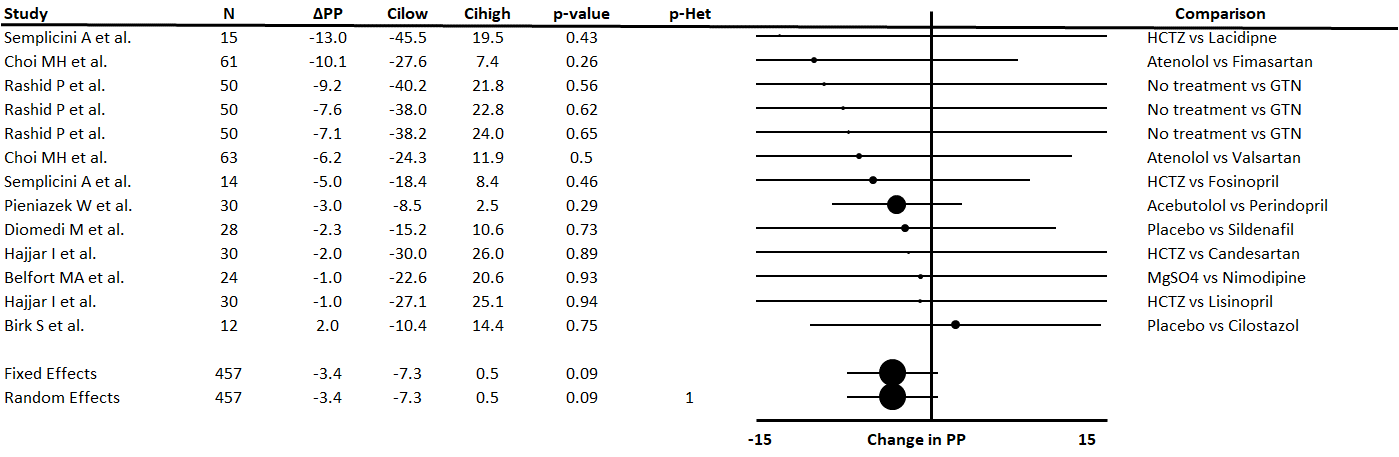


**Supplemental Figure 10. Forest plot of difference in systolic blood pressure (SBP), diastolic blood pressure (DBP), cerebral mean flow velocity (MFV) or cerebral arterial pulsatility index (PI) between groups randomised to a vasodilating medication versus a non-vasodilating control group, limited to studies reporting both BP changes and ultrasound indices.**  Control groups included patients treated with either placebo, a non-vasodilating medication or no change in treatment, with effect sizes combined by both fixed effects and random effects meta-analysis weighted by the inverse variance. Cilow- confidence interval lower limit; Cihigh confidence interval upper limit; p-het – p value for heterogeneity; N – number of subjects; GTN – glyceryl trinitrate; HCTZ – hydrochlorothiazide;

1. SBP

1. DBP

1. Mean flow velocity

1. Pulsatility Index

**Supplemental Figure 11. Meta-regression of change in standardised mean differences in mean flow velocity (SMD-MFV), systolic blood (SMD-SBP), diastolic blood pressure (SMD-DBP) and cerebral arterial pulsatility index (SMD-PI).** Regressions are weighted by the inverse variance calculated for the meta-analyses.


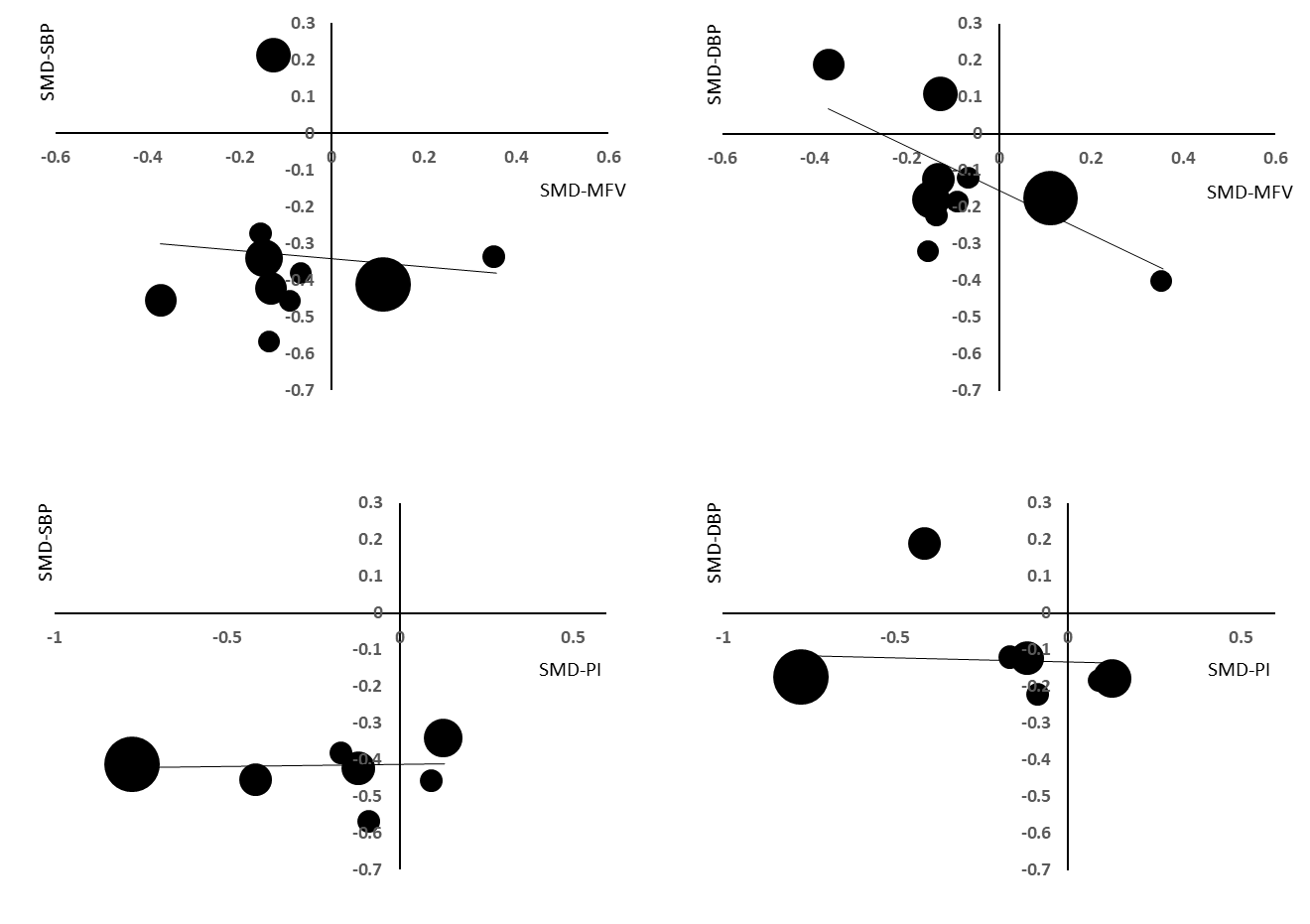


**Supplemental Table 1. Characteristics of Included Studies**

|  | 1st Author | Year | N | Age | Randomised | Blinding | Design | Placebo | Dosing | Population | Comparison |
| --- | --- | --- | --- | --- | --- | --- | --- | --- | --- | --- | --- |
| 1 | Schulz JM | 2018 | 10 | 22 | No | Single | Crossover | Placebo | Single | Healthy | Placebo vs Nitroglycerin |
| 2 | Choi MH | 2018 | 95 | 61 | Randomised | Double | Parallel | No Placebo | Prolonged | Stroke | Atenolol vs Valsartan vs Fimasartan |
| 3 | Lindberg U | 2017 | 17 | 35 | Randomised | Double | Crossover | Placebo | Prolonged | Becker MD | Placebo vs Sildenafil |
| 4 | Zhang W | 2016 | 610 | 62 | Randomised | Open | Parallel | No Placebo | Prolonged | Stroke | Control (no tx) vs Vinpocetine |
| 5 | Wong RH | 2016 | 36 | 69 | Randomised | Double | Crossover | Placebo | Single | T2DM | Placebo vs Resveratol vs Resveratol |
| 6 | Han SW | 2014 | 203 | 65 | Randomised | Double | Parallel | Placebo | Prolonged | Stroke | Placebo vs Cilostazol |
| 7 | Tzeng YC | 2013 | 23 | 26 | Randomised | Open | Parallel | Placebo | Single | Healthy | Placebo vs Nimodipine |
| 8 | Sakurai H | 2013 | 20 | 79 | Randomised | Open | Parallel | No Placebo | Prolonged | Dementia | Control (ASA or Clopidogrel) vs Cilostazol |
| 9 | Beer C | 2012 | 52 | 69 | Randomised | Double | Parallel | Placebo | Prolonged | Stroke | Placebo vs Irbesartan |
| 10 | Kume K | 2012 | 20 | 79 | Randomised | Open | Parallel | No Placebo | Prolonged | Dementia | Amlodipine vs Telmisartan |
| 11 | Kennedy DO | 2010 | 24 | 20 | Randomised | Single | Crossover | Placebo | Single | Healthy | Placebo vs trans-resveratol vs trans-resveratol |
| 12 | Hong KS | 2010 | 196 | 62 | Randomised | Double | Parallel | No Placebo | Prolonged | Stroke | Cilnidipine vs Losartan |
| 13 | Matsumoto S | 2009 | 35 | 61 | Randomised | Double | Parallel | No Placebo | Prolonged | Stroke | Amlodipine vs Olmesartan |
| 14 | Rashid P | 2003 | 90 | 71 | Randomised | Open | Parallel | No Placebo | Single | Stroke | No tx vs GTN vs GTN |
| 15 | Rosengarten | 2006 | 11 | 49 | No | Open | Crossover | No Placebo | Single | PAH | Iloprost vs Sildenafil |
| 16 | Fu CH | 2005 | 90 | 60 | No | Open | Parallel | No Placebo | Prolonged | HTN | Control vs Nifedipine SR vs Atenolol |
| 17 | Diomedi M | 2005 | 28 | 57 | Randomised | Double | Parallel | Placebo | Single | Healthy | Placebo vs Sildenafil |
| 18 | Koksal M | 2005 | 30 | 46 | No | Double | Parallel | Placebo | Single | Healthy | Placebo vs Sildenafil |
| 19 | Birk S | 2004 | 12 | 26 | Randomised | Double | Crossover | Placebo | Single | Healthy | Placebo vs Cilostazol |
| 20 | Nazir FS | 2005 | 25 | 68 | Randomised | Double | Parallel | Placebo | Single | Stroke | Placebo vs Perindopril |
| 21 | Hatazawa J | 2004 | 19 | 65 | Randomised | Single | Parallel | Placebo | Prolonged | Stroke | Placebo vs Perindopril |
| 22 | Walters M | 2004 | 12 | 63 | Randomised | Double | Crossover | Placebo | Prolonged | Stroke | Placebo vs Perindopril |
| 23 | Arnavaz A | 2003 | 6 | 26 | Randomised | Double | Crossover | Placebo | Single | Healthy | Placebo vs Sildenafil |
| 24 | Pieniazek W | 2001 | 30 | 47 | Randomised | Double | Crossover | No Placebo | Prolonged | Hypertension | Acebutolol vs Perindopril |
| 25 | Semplicini A | 2000 | 15 | ~50 | Randomised | Double | Parallel | Placebo | Prolonged | Hypertension | HCTZ vs Lacidipne |
| 26 | Kruuse C | 2000 | 10 | 25 | Randomised | Double | Crossover | Placebo | Single | Healthy | PlAcebo vs Pentoxifylline |
| 27 | Belfort MA | 1999 | 21 | 27 | Randomised | Open | Parallel | No Placebo | Single | PET | MgSO4 vs Nimodipine |
| 28 | Semplicini A | 1993 | 14 | 61 | Randomised | Double | Parallel | No Placebo | Prolonged | HTN | HCTZ vs Fosinopril |
| 29 | Traub YM | 1991 | 20 | 67 | Randomised | Single | Crossover | No Placebo | Prolonged | Hypertension | Enalapril vs Nifedipine l |
| 30 | Kraaier V | 1991 | 24 | 23 | Randomised | Double | Crossover | Placebo | Prolonged | ischaemia) | Placebo vs Nimodipine |
| 31 | James IM | 1978 | 10 | 70 | No | Double | Parallel | Placebo | Prolonged | Dementia | Placebo vs Naftidofuryl |
| 32 | Hajjar I | 2014 | 47 | 72 | Randomised | Double | Parallel | No Placebo | Prolonged | MCI | HCTZ vs Candesartan vs Lisinopril |
| 33 | Webb AJS | 2016 | 10 | 29 | Randomised | Single | Crossover | No Placebo | Prolonged | Healthy | Propranolol LA vs Amlodipine |
| 34 | Han SW | 2013 | 203 | 65 | Randomised | Double | Parallel | Placebo | Prolonged | Stroke | Placebo vs Cilostazol |
| 35 | Willmot M | 2006 | 18 | 70 | Randomised | Single | Parallel | Placebo | Prolonged | Stroke | Placebo vs GTN |
| 36 | Guo JJ | 2009 | 68 | 60 | Randomised | Open | Parallel | No Placebo | Prolonged | Stroke | Aspirin vs Cilostazol |

**References**

| 1. Schulz JM, Al-Khazraji BK, Shoemaker JK. Sodium nitroglycerin induces middle cerebral artery vasodilatation in young, healthy adults. Exp Physiol. 2018 Aug;103(8):1047-1055. doi: 10.1113/EP087022. Epub 2018 Jun 13. PubMed PMID: 29766604; PubMed Central PMCID: PMC6099468. |
| --- |
| 2. Choi MH, Lee JS, Lee SE, Lee SJ, Yoon D, Park RW, Hong JM. Central and cerebral haemodynamic changes after antihypertensive therapy in ischaemic stroke patients: A double-blind randomised trial. Sci Rep. 2018 Jan 24;8(1):1556. doi: 10.1038/s41598-018-19998-4. PubMed PMID: 29367614; PubMed Central PMCID: |
| 3. Lindberg U, Witting N, Jørgensen SL, Vissing J, Rostrup E, Larsson HB, Kruuse C. Effects of Sildenafil on Cerebrovascular Reactivity in Patients with Becker Muscular Dystrophy. Neurotherapeutics. 2017 Jan;14(1):182-190. doi: 10.1007/s13311-016-0467-x. PubMed PMID: 27485237; PubMed Central PMCID: |
| 4. Zhang W, Huang Y, Li Y, Tan L, Nao J, Hu H, Zhang J, Li C, Kong Y, Song Y. Efficacy and Safety of Vinpocetine as Part of Treatment for Acute Cerebral Infarction: A Randomized, Open-Label, Controlled, Multicenter CAVIN (Chinese Assessment for Vinpocetine in Neurology) Trial. Clin Drug Investig. 2016 |
| 5. Wong RH, Nealon RS, Scholey A, Howe PR. Low dose resveratrol improves cerebrovascular function in type 2 diabetes mellitus. Nutr Metab Cardiovasc Dis. 2016 May;26(5):393-9. doi: 10.1016/j.numecd.2016.03.003. Epub 2016 Mar 14. PubMed PMID: 27105868. |
| 6. Han SW, Song TJ, Bushnell CD, Lee SS, Kim SH, Lee JH, Kim GS, Kim OJ, Koh IS, Lee JY, Suk SH, Lee SI, Nam HS, Kim WJ, Lee KY, Park JH, Kim JY, Park JH. Cilostazol decreases cerebral arterial pulsatility in patients with mild white matter hyperintensities: subgroup analysis from the Effect of Cilostazol in Acute |
| 7. Tzeng YC, MacRae BA. Interindividual relationships between blood pressure and cerebral blood flow variability with intact and blunted cerebrovascular control. J Appl Physiol (1985). 2013 Apr;114(7):888-95. doi: 10.1152/japplphysiol.01388.2012. Epub 2013 Jan 31. PubMed PMID: 23372142. |
| 8. Sakurai H, Hanyu H, Sato T, Kume K, Hirao K, Kanetaka H, Iwamoto T. Effects of cilostazol on cognition and regional cerebral blood flow in patients with Alzheimer's disease and cerebrovascular disease: a pilot study. Geriatr Gerontol Int. 2013 Jan;13(1):90-7. doi: 10.1111/j.1447-0594.2012.00866.x. Epub 2012 Jun 4. |
| 9. Beer C, Blacker D, Bynevelt M, Hankey GJ, Puddey IB. A randomized placebo controlled trial of early treatment of acute ischemic stroke with atorvastatin and irbesartan. Int J Stroke. 2012 Feb;7(2):104-11. doi: 10.1111/j.1747-4949.2011.00653.x. Epub 2011 Nov 2. PubMed PMID: 22044557. |
| 10. Kume K, Hanyu H, Sakurai H, Takada Y, Onuma T, Iwamoto T. Effects of telmisartan on cognition and regional cerebral blood flow in hypertensive patients with Alzheimer's disease. Geriatr Gerontol Int. 2012 Apr;12(2):207-14. doi: 10.1111/j.1447-0594.2011.00746.x. Epub 2011 Sep 19. PubMed PMID: 21929736. |
| 11. Kennedy DO, Wightman EL, Reay JL, Lietz G, Okello EJ, Wilde A, Haskell CF. Effects of resveratrol on cerebral blood flow variables and cognitive performance in humans: a double-blind, placebo-controlled, crossover investigation. Am J Clin Nutr. 2010 Jun;91(6):1590-7. doi: 10.3945/ajcn.2009.28641. Epub 2010 Mar 31. |
| 12. Hong KS, Kang DW, Bae HJ, Kim YK, Han MK, Park JM, Rha JH, Lee YS, Koo JS, Cho YJ, Kwon SU, Kim SE, Park SH. Effect of cilnidipine vs losartan on cerebral blood flow in hypertensive patients with a history of ischemic stroke: a randomized controlled trial. Acta Neurol Scand. 2010 Jan;121(1):51-7. doi: |
| 13. Matsumoto S, Shimodozono M, Miyata R, Kawahira K. Benefits of the angiotensin II receptor antagonist olmesartan in controlling hypertension and cerebral hemodynamics after stroke. Hypertens Res. 2009 Nov;32(11):1015-21. doi: 10.1038/hr.2009.143. Epub 2009 Sep 11. PubMed PMID: 19745828. |
| 14. Rashid P, Weaver C, Leonardi-Bee J, Bath F, Fletcher S, Bath P. The effects of transdermal glyceryl trinitrate, a nitric oxide donor, on blood pressure, cerebral and cardiac hemodynamics, and plasma nitric oxide levels in acute stroke. J Stroke Cerebrovasc Dis. 2003 May-Jun;12(3):143-51. PubMed PMID: |
| 15. Rosengarten B, Schermuly RT, Voswinckel R, Kohstall MG, Olschewski H, Weissmann N, Seeger W, Kaps M, Grimminger F, Ghofrani HA. Sildenafil improves dynamic vascular function in the brain: studies in patients with pulmonary hypertension. Cerebrovasc Dis. 2006;21(3):194-200. Epub 2005 Dec 23. PubMed PMID: |
| 16. Fu CH, Yang CC, Kuo TB. Effects of different classes of antihypertensive drugs on cerebral hemodynamics in elderly hypertensive patients. Am J Hypertens. 2005 Dec;18(12 Pt 1):1621-5. PubMed PMID: 16364836. |
| 17. Diomedi M, Sallustio F, Rizzato B, Ferrante F, Leone G, Spera E, Scarfini M, Bernardi G. Sildenafil increases cerebrovascular reactivity: a transcranial Doppler study. Neurology. 2005 Sep 27;65(6):919-21. Epub 2005 Jul 28. PubMed PMID: 16051646. |
| 18. Koksal M, Ozdemir H, Kargi S, Yesilli C, Tomaç S, Mahmutyazicioglu K, Mungan A. The effects of sildenafil on ocular blood flow. Acta Ophthalmol Scand. 2005 Jun;83(3):355-9. PubMed PMID: 15948790. |
| 19. Birk S, Kruuse C, Petersen KA, Jonassen O, Tfelt-Hansen P, Olesen J. The phosphodiesterase 3 inhibitor cilostazol dilates large cerebral arteries in humans without affecting regional cerebral blood flow. J Cereb Blood Flow Metab. 2004 Dec;24(12):1352-8. PubMed PMID: 15625409. |
| 20. Nazir FS, Overell JR, Bolster A, Hilditch TE, Lees KR. Effect of perindopril on cerebral and renal perfusion on normotensives in mild early ischaemic stroke: a randomized controlled trial. Cerebrovasc Dis. 2005;19(2):77-83. Epub 2004 Dec 17. PubMed PMID: 15608430. |
| 21. Hatazawa J, Shimosegawa E, Osaki Y, Ibaraki M, Oku N, Hasegawa S, Nagata K, Hirata Y, Miura Y. Long-term angiotensin-converting enzyme inhibitor perindopril therapy improves cerebral perfusion reserve in patients with previous minor stroke. Stroke. 2004 Sep;35(9):2117-22. Epub 2004 Jul 15. PubMed PMID: 15256675. |
| 22. Walters M, Muir S, Shah I, Lees K. Effect of perindopril on cerebral vasomotor reactivity in patients with lacunar infarction. Stroke. 2004 Aug;35(8):1899-902. Epub 2004 May 27. PubMed PMID: 15166388. |
| 23. Arnavaz A, Aurich A, Weissenborn K, Hartmann U, Emrich HM, Schneider U. Effect of sildenafil (Viagra) on cerebral blood flow velocity: a pilot study. Psychiatry Res. 2003 Apr 1;122(3):207-9. PubMed PMID: 12694895. |
| 24. Pieniazek W, Dimitrow PP, Jasiński T. Comparison of the effect of perindopril and acebutolol on cerebral hemodynamics in hypertensive patients. Cardiovasc Drugs Ther. 2001 Jan;15(1):63-7. PubMed PMID: 11504165. |
| 25. Semplicini A, Maresca A, Simonella C, Carollo C, Chierichetti F, Santipolo N, Pauletto P, Ferlin G, Pessina AC. Cerebral perfusion in hypertensive patients: effects of lacidipine and hydrochlorothiazide. J Cardiovasc Pharmacol. 2000;35(3 Suppl 1):S13-8. PubMed PMID: 11347856. |
| 26. Kruuse C, Jacobsen TB, Thomsen LL, Hasselbalch SG, Frandsen EK, Dige-Petersen H, Olesen J. Effects of the non-selective phosphodiesterase inhibitor pentoxifylline on regional cerebral blood flow and large arteries in healthy subjects. Eur J Neurol. 2000 Nov;7(6):629-38. PubMed PMID: 11136348. |
| 27. Belfort MA, Saade GR, Yared M, Grunewald C, Herd JA, Varner MA, Nisell H. Change in estimated cerebral perfusion pressure after treatment with nimodipine or magnesium sulfate in patients with preeclampsia. Am J Obstet Gynecol. 1999 Aug;181(2):402-7. PubMed PMID: 10454691. |
| 28. Semplicini A, Simonella C, Meneghetti G, Chierichetti F, Serena L, Claroni F, Fazari G, Ferlin G, Pessina AC. Effects of fosinopril and hydrochlorothiazide on cerebral perfusion in uncomplicated essential hypertension. J Hypertens Suppl. 1993 Dec;11(5):S372-3. PubMed PMID: 8158428. |
| 29. Traub YM, Ayache AS, Groshar D. The effect of enalapril and calcium antagonists on blood pressure and cerebral perfusion in elderly hypertensives. J Hypertens Suppl. 1991 Dec;9(6):S378-9. PubMed PMID: 1819000. |
| 30. Kraaier V, van Huffelen AC, Wieneke GH, Hesselink JM. Nimodipine tested in a human model of cerebral ischaemia. Electroencephalographic and transcranial Doppler ultrasound investigations in normal subjects during standardized hyperventilation. Eur J Clin Pharmacol. 1991;40(1):17-21. PubMed PMID: 2060540. |
| 31. James IM, Newbury P, Woolard ML. The effect of naftidrofuryl oxalate on cerebral blood flow in elderly patients. Br J Clin Pharmacol. 1978 Dec;6(6):545-6. PubMed PMID: 728328; PubMed Central PMCID: PMC1429701. |
| 32. Hajjar I, Hart M, Chen YL, Mack W, Novak V, C Chui H, Lipsitz L. Antihypertensive therapy and cerebral hemodynamics in executive mild cognitive impairment: results of a pilot randomized clinical trial. J Am Geriatr Soc. 2013 Feb;61(2):194-201. doi: 10.1111/jgs.12100. Epub 2013 Jan 25. PubMed PMID: |
| 33. Webb AJ, Rothwell PM. Magnetic Resonance Imaging Measurement of Transmission of Arterial Pulsation to the Brain on Propranolol Versus Amlodipine. Stroke. 2016 Jun;47(6):1669-72. doi: 10.1161/STROKEAHA.115.012411. Epub 2016 May 3. PubMed PMID: 27143273; PubMed Central PMCID: PMC5328287. |
| 34. Willmot M, Ghadami A, Whysall B, Clarke W, Wardlaw J, Bath PM. Transdermal glyceryl trinitrate lowers blood pressure and maintains cerebral blood flow in recent stroke. Hypertension. 2006 Jun;47(6):1209-15. Epub 2006 May 8. PubMed PMID: 16682611. |
| 35. Han SW, Lee SS, Kim SH, Lee JH, Kim GS, Kim OJ, Koh IS, Lee JY, Suk SH, Lee SI, Nam HS, Kim WJ, Yong SW, Lee KY, Park JH. Effect of cilostazol in acute lacunar infarction based on pulsatility index of transcranial Doppler (ECLIPse): a multicenter, randomized, double-blind, placebo-controlled trial. Eur Neurol. |
| 36. Guo JJ, Xu E, Lin QY, Zeng GL, Xie HF. Effect of cilostazol on cerebral arteries in secondary prevention of ischemic stroke. Neurosci Bull. 2009 Dec;25(6):383-90. doi: 10.1007/s12264-009-6192-2. PubMed PMID: 19927175; PubMed Central PMCID: PMC5552506. |

**Search Strategy (Pubmed)**

("transcranial ultrasound"[Title/Abstract] OR "transcranial doppler"[Title/Abstract] OR "transcranial ultrasonography"[Title/Abstract] OR "Gosling's pulsatility index"[Title/Abstract] OR "pulsatility index"[Title/Abstract] OR "peak systolic velocity"[Title/Abstract] OR "end diastolic velocity"[Title/Abstract] OR "mean flow velocity"[Title/Abstract] OR "cerebral blood flow"[Title/Abstract] OR "cerebral perfusion"[Title/Abstract] OR "ASL"[Title/Abstract] OR "CT perfusion"[Title/Abstract] OR "MRI perfusion"[Title/Abstract] OR "MR perfusion"[Title/Abstract] OR "cerebrovascular reactivity"[Title/Abstract] OR "breath holding index"[Title/Abstract] OR "hyperventilation"[Title/Abstract] OR "cerebral perfusion"[Title/Abstract]) AND (vasodilator[Title/Abstract] OR vasodilation[Title/Abstract] OR vasodilating[Title/Abstract] OR endothelin[Title/Abstract] OR phosphodiesterase[Title/Abstract] OR "calcium channel blocker"[Title/Abstract] OR angiotensin[Title/Abstract] OR "nitric oxide"[Title/Abstract] OR "nitrous oxide"[Title/Abstract] OR "amlodipine"[Title/Abstract] OR "felodipine"[Title/Abstract] OR "nimodipine"[Title/Abstract] OR "nitrendipine"[Title/Abstract] OR "isradipine"[Title/Abstract] OR "lacidipine"[Title/Abstract] OR "lercanidipine"[Title/Abstract] OR "nicardipine"[Title/Abstract] OR "nifedipine"[Title/Abstract] OR "nitrendipine"[Title/Abstract] OR "sildenafil"[Title/Abstract] OR "tadalafil"[Title/Abstract] OR "vardenafil"[Title/Abstract] OR "cilostazol"[Title/Abstract] OR "amrinone"[Title/Abstract] OR "candesartan"[Title/Abstract] OR eprosartan[Title/Abstract] OR irbesartan[Title/Abstract] OR losartan[Title/Abstract] OR olmesartan[Title/Abstract] OR telmisartan[Title/Abstract] OR valsartan[Title/Abstract] OR captopril[Title/Abstract] OR cilazapril[Title/Abstract] OR enalapril[Title/Abstract] OR fosinopril[Title/Abstract] OR imidapril[Title/Abstract] OR lisinopril[Title/Abstract] OR moexipril[Title/Abstract] OR perindopril[Title/Abstract] OR quinapril[Title/Abstract] OR ramipril[Title/Abstract] OR ambrisentan[Title/Abstract] OR bosentan[Title/Abstract] OR diazoxide[Title/Abstract] OR hydralazine[Title/Abstract] OR vinpocetine[Title/Abstract] OR sitaxsentan[Title/Abstract] OR iloprost[Title/Abstract] OR minoxidil[Title/Abstract] OR naftidrofuryl[Title/Abstract] OR nebivolol[Title/Abstract] OR nicorandil[Title/Abstract])

**Inclusion Criteria**

1. Controlled comparison
2. Compares groups of similar individuals
3. Control groups include placebo, no treatment or a non-vasodilating medication
4. Experimental group uses an accepted vasodilators:
   1. Phosphodiesterase inhibitors (specifically 3 or 5)
   2. Calcium channel blockers
   3. Renin-angiotensin system inhibitors (ACEI, ARBs etc)
   4. Endothelin receptor antagonists
   5. Nitric oxide donrs
   6. Direct vasodilators (ie hydralazine)
   7. Alpha-blockers
   8. Nebivolol, nicorandil
   9. Agents with a likely vasodilator effect: Resveratrol, vinpocetine
5. Acute treatment (within 7 days of an event) or chronic treatment (> 7 days after an acute event, or use in a chronic condition)
6. In any patient group (healthy individuals, stroke patients, small vessel disease or cognitive disorders…)
7. Reports effects on cerebral haemodynamics or reactivity to carbon dioxide using either:
   1. Transcranial Doppler ultrasound
   2. MRI sequences sensitive to blood flow or blood volume (BOLD, ASL, contrast based perfusion, phase contrast)
   3. CT perfusion
   4. Near infrared spectroscopy
   5. Any direct measure of cerebral blood flow (PET, SPECT etc)
8. Reports numerical effect size

**Exclusion Criteria**

1. Post-SAH vasospasm
2. Moderate to Severe head injury
3. Underlying genetic disorder (ie CADASIL)
4. Sequential study design with consistent treatment order (crossover trials with randomised order of interventions allowed)
